# Economic and disease burden of RSV-associated hospitalizations in young children in France, from 2010 through 2018

**DOI:** 10.1186/s12879-021-06399-8

**Published:** 2021-08-02

**Authors:** C. Demont, N. Petrica, I. Bardoulat, S. Duret, L. Watier, A. Chosidow, M. Lorrot, A. Kieffer, M. Lemaitre

**Affiliations:** 1 Sanofi Pasteur, 14 Espace Henry Vallée, 69007 Lyon, France; 2 IQVIA, 92400 Courbevoie, France; 3 Université Paris-Saclay, UVSQ, Inserm, CESP, 94807 Villejuif, France; 4 Department of Pediatrics, Armand Trousseau Hospital (AP-HP), 75012 Paris, France

**Keywords:** Respiratory syncytial virus, Hospitalization, Socio-economic impact, Burden, Children

## Abstract

**Background:**

Respiratory syncytial virus (RSV) is the main cause of infant and child hospitalizations. The study objective is to estimate the RSV-associated hospitalizations and economic burden in young children in France to inform future preventive strategies.

**Methods:**

We conducted a retrospective analysis of RSV-associated hospitalizations data from the French Hospital database (PMSI-MCO) which covers the entire French population. All children aged < 5 years hospitalized with RSV ICD-10 codes (J210, J219, J45, J121, J205, R062) from 2010 to 2018, were included. Descriptive analyses were conducted by RSV seasons (Oct to March), by respiratory years (July to June) and per age groups.

**Results:**

On average 45,225 RSV-associated hospitalizations (range: 43,715 – 54,616) per season was reported in France, 69% among children < 1 year old. This represents 28% of all-cause hospitalizations that occurred among children < 1 year old, and less than 10% of all-cause hospitalizations in older children. Number of RSV-associated hospitalizations were similar for infants born during (Oct-March) or outside (April–September) their first RSV season. The highest risk being reported for infants born from September through November. The associated hospitalization cost increased between 2010 - 11 and 2017–18, from €93.2 million to €124.1 million, respectively, and infants < 1 year old represented 80% of the economic burden.

**Conclusion:**

RSV is an important cause of child hospitalization in France. The burden on healthcare system is mainly driven by < 1 year olds, and preventive strategies should be implemented before the first RSV season.

**Supplementary Information:**

The online version contains supplementary material available at 10.1186/s12879-021-06399-8.

## Introduction

Respiratory Syncytial Virus (RSV) has been recognized as a major cause of lower respiratory tract infections (LRTI) in children [1] and is the leading cause of hospital admissions in < 1 year olds [2, 3]. Risk of hospital admissions due to RSV is higher in children with known risk factors (prematurity, comorbidities, age < 6 months, birth proximity to RSV season) [1, 3, 4]. Nevertheless, severe outcomes requiring hospital admission often occur in previously healthy children [5]. Treatment is mainly based on supportive care and the monoclonal antibody palivizumab is the only available prophylactic strategy indicated in restricted at-risk pediatric populations to prevent RSV-related complications, including hospitalizations [6, 7].

In Northern Europe, respiratory tract infections associated with RSV generally occur between October and March, with a peak of infection in France around November/December [8]. Every year, French surveillance data suggests that 30% of children aged < 2 years have bronchiolitis, mostly due to RSV, of which 2–3% are hospitalized and less than 1% leads to death [9]. In 2009, a national French study reported an overall incidence of hospital admissions for bronchiolitis of 35.8 per 1000 children < 1 year old [10]. In 2018, in a single centre study by Kramer et al., the incidence of hospital admissions for RSV infection during the first year of life was estimated to be 14.5 per 1000 births [11].

RSV clinical burden is associated with high direct costs related to healthcare consumption. To our knowledge, the economic burden of RSV related hospitalizations in France was only recently assessed by Kramer et al., the mean cost for RSV hospitalization was estimated to €3,973 per child < 1 year old [11]. In a recent US study, a wide range of annual costs based on risk was observed from $8,000 up to $40,000 [12].

With the approaching launch of new and innovative immunization strategies, such as maternal vaccination or monoclonal antibodies, it has become highly relevant to provide contemporary data on RSV disease burden and healthcare associated cost in countries where these informations are lacking, such as France. The uniqueness of French national health insurance covering 67 million inhabitants is an opportunity to evaluate and update RSV disease burden at hospital [13].

The aim of this study was to provide detailed information on clinical and economic burden at a population level of RSV-associated hospitalization in children aged < 5 years in France between 2010 and 2018, with a specific focus on children < 1 year old. This will provide the background evidence related to RSV disease burden before immunization strategies become available.

## Methods

### Data sources

Data were extracted from the French national hospital discharge database (*Programme de Médicalisation des Systèmes d’Information – PMSI*), covering the whole population [13]. PMSI provides medico-administrative information on secondary and tertiary care from the public and private sectors as described previously [14]. Study was carried out in compliance with the French regulatory [15].

- *Hospitalization data*

All RSV-associated hospital stays in general medicine, surgery and obstetric medical facilities (PMSI-MSO) as well as all-cause hospitalization from 2010 through 2018 in children aged < 5 years were selected for this analysis. RSV-associated hospitalization were identified using the following International Classification of Diseases 10th revision (ICD-10) codes as the primary diagnosis (PD): J210 (acute bronchiolitis due to RSV), J219 (acute bronchiolitis, unspecified), J121 (pneumonia due to RSV), J205 (bronchitis due to RSV), J45 (asthma) and R062 (wheezing).

Hospital admissions with an ICD-10 code of bronchiolitis (J210 or J219 ICD-10 codes) recorded as an associated diagnosis (AD) were also selected when the primary diagnosis was a “disorder of the respiratory system” (J00-J99 ICD-10 codes) or “certain conditions originating in the perinatal period” (P00-P96 ICD-10 codes), assuming that RSV-associated bronchiolitis contributed to the hospitalization despite not being listed as primary diagnosis.

b) Demographic data

Monthly births recorded and published by the National Institute of Statistics and Economic Studies (INSEE) were used to estimate the incidence of RSV in France for the 2010–2018 period [16].

### Statistical analyses

A descriptive analysis of RSV-associated hospitalizations during RSV seasons (defined from October to March the following year) was carried out over the period 2010–2018 [17].

Analyses were stratified by age group, defined by chronological ages (< 1 year old vs. ≥1 year old) and further stratified by month categories and by gestational weeks at birth (weeks’ gestational age, wGA).

wGA was determined based on mother’s information on the number of weeks of amenorrhea, directly available in PMSI database. Data on mother’s amenorrhea was not consistently available, especially for records of children born before 2009. Full term birth was defined as birth after 36 weeks of amenorrhea, otherwise it was considered as preterm birth, for which subcategories were defined: extremely preterm birth (< 29 weeks), very preterm birth (29–32 weeks) and moderate preterm birth (33–35 weeks). The stratification was based on the palivizumab recommendations of the healthcare authorities and the society of neonatology in France [7].

c) *Epidemiological analysis*

RSV-associated hospitalization incidence was estimated by the number of RSV-associated hospitalizations per 1000 person-months for each RSV season. Total time of exposure was calculated from demographic data, corresponding to the number of months during which children in specific age subgroups were at risk for RSV, during the season. Incidence was also estimated for the respiratory year, defined from July to June the following year to ensure that an entire RSV season was covered.

We assessed if the month of birth has an impact on the risk of RSV hospitalization. For children born during a given month, the number of RSV-associated hospitalizations was computed from birth to the end of their first RSV season. The RSV-associated hospitalization risk across the 8 RSV seasons was determined by dividing the total number of RSV-associated hospitalisations from the total number of children born during the same month. For this risk estimation, a more restrictive definition of RSV-associated hospitalizations was considered excluding J45 (asthma) and R062 (wheezing).

Children characteristics were described considering the age, the gender and the known risk factors for RSV-associated hospitalization: presence or absence of congenital heart defects (PD, related diagnosis (RD) or AD ICD-10 Q20-Q26 in hospital discharge), bronchopulmonary dysplasia (PD, RD or AD ICD-10 P27.1), Down syndrome (PD, RD or AD ICD-10 Q90), cystic fibrosis with pulmonary manifestations (PD, RD or AD ICD-10 Q90, E84).

All-cause hospital admissions occurring within 3 months following the initial RSV-associated hospitalization were identified. The number and proportion of patients affected by re-hospitalization and the average number of re-hospitalizations per patient were estimated.

d) *Economic analysis*

Direct cost of RSV burden was calculated based on the resources used during RSV-associated hospitalizations recorded in PMSI database. Total cost and average cost per stay per patient were calculated by RSV season from a collective perspective, considering the French national health insurance reimbursement, medical professionals costs and running costs of the medical unit (e.g. cleaning and laundry), public complementary and private insurance and out-of-pocket charges.

These costs were calculated for all RSV seasons according to the DRG (Diagnosis Related Group) based payment system for reimbursement of acute care (MSO). The costs were adjusted to 2018 euro value by applying the price index of health products in mainland France [16].

## Results

### Epidemiologic burden of RSV

#### Hospital stays and patients’ characteristics

A total of 407,025 RSV-associated hospitalizations were identified over 8 RSV seasons in children aged < 5 years (Table 1). A 15%-increase in RSV-associated hospitalization was observed between 2010/11 (*N* = 43,715) and 2013/14 (*N* = 50,373), and 8% between 2014/15 (N = 50,728) and 2017/18 (*N* = 54,616). During the 8-year period, RSV-associated hospitalizations among all-cause hospitalizations increased from 22 to 28% in children < 1 year old during RSV seasons and from 13 to 16% during respiratory years (Table S1).

**Table 1:** Number of RSV hospital stays, according to age groups and RSV seasons from 2010 through 2018 in France

		Age group (month old)					
RSV season/ N	All ages	< 3	3–5	6–11	12–23	24–35	36–59
2010/2011	43,715	13,691	8146	8098	7026	3105	3649
2011/2012	47,973	15,351	8665	8888	7730	3482	3857
2012/2013	50,949	17,515	9191	8562	7576	3658	4447
2013/2014	50,373	17,280	9476	8551	7418	3471	4177
2014/2015	50,728	16,572	8735	8604	7472	4071	5274
2015/2016	54,585	18,873	10,071	9236	7918	3837	4650
2016/2017	54,086	18,102	10,127	9397	8109	3877	4474
2017/2018	54,616	18,734	10,458	9492	7752	3703	4477
Mean annual number	**50,878**	**17,015**	**9359**	**8854**	**7625**	**3651**	**4376**

*RSV* Respiratory syncytial virus

**Fig. 1:**
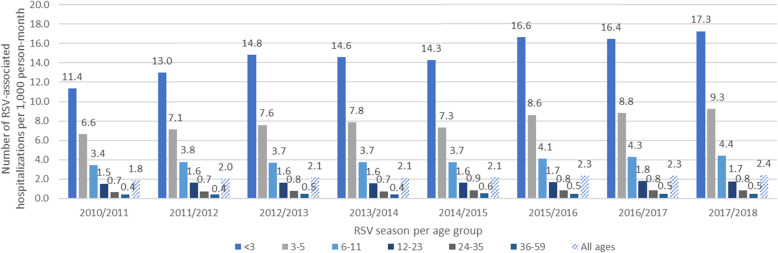
Rate of RSV-associated hospitalizations, per 1000 person-month, by RSV season and age group, from 2010 through 2018 in France

RSV-associated hospitalization incidence varied from 1.83 to 2.40 per 1000 person-months (Fig. 1). Incidence in the < 1-year old population increased from 6.23 to 8.85 per 1000 person-months, representing 69% of RSV-associated hospitalizations. RSV-associated hospitalizations of children < 1 year old were mainly coded as acute bronchiolitis due to RSV (J210–45%) and acute bronchiolitis - unspecified (J219–48%) (Fig. S1). In older children, asthma (J45) was the main code used for hospital admission (70%), especially for children ≥2 years old (89%).

**Fig. 2:**
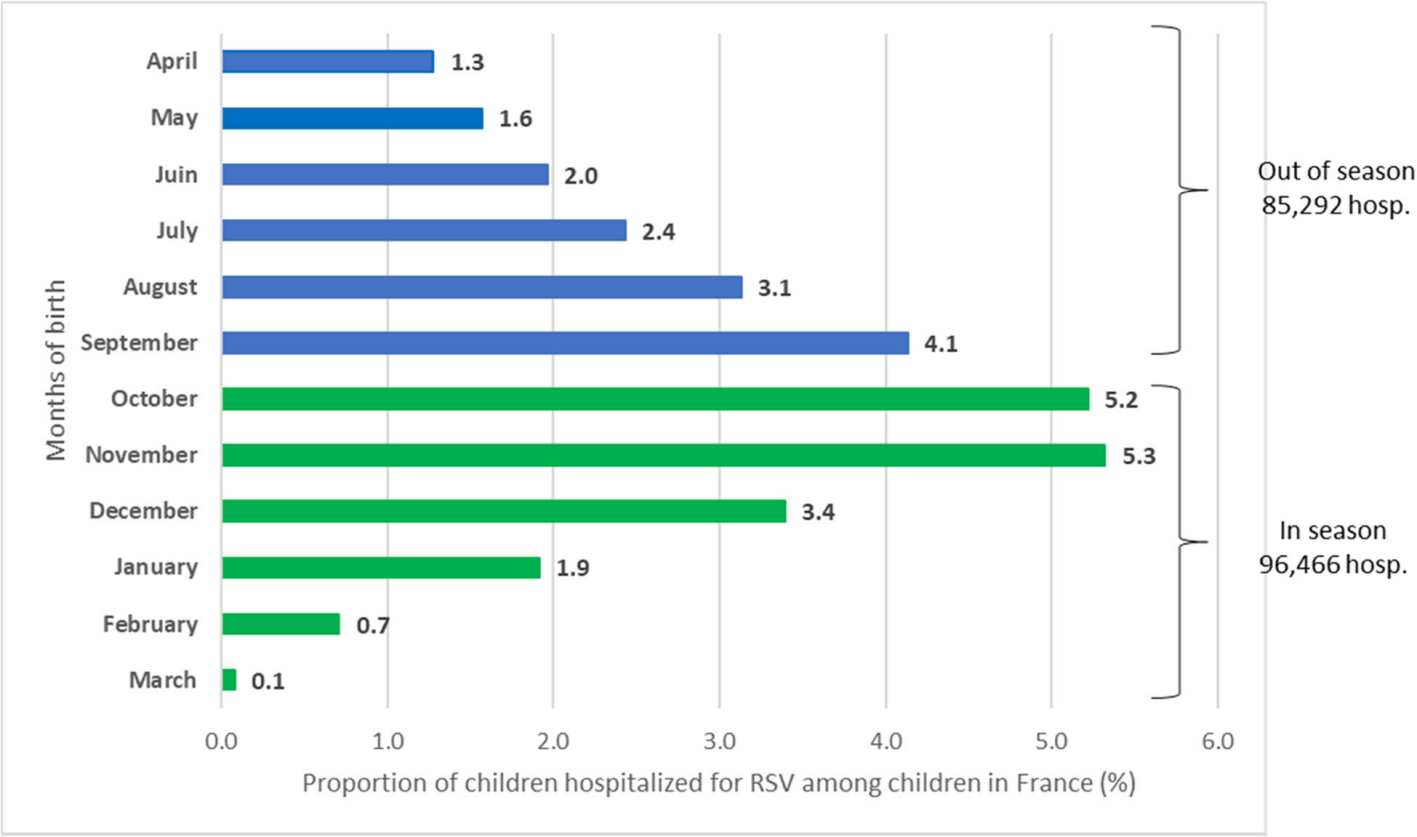
Impact of the month of birth on the risk of RSV-associated hospitalization (RSV-specific ICD-10 codes) from 2010 through 2018 in France

The risk of RSV-associated hospitalization, during their first RSV season exposure, increased for children born from April up to November (Fig. 2). Children born between September and November were at the highest risk to be hospitalized for RSV (4.1 and 5.3% respectively) compared to other months of birth. A decreased risk of RSV-associated hospitalization was observed for children born from December up to March (3.4 and 0.1% respectively). The total number of RSV-associated hospitalizations was comparable between those born outside (April – September) or during RSV season (October – March) with 85,292 versus 96,466 RSV-associated hospitalization over the 8-year period.

Table 2 represents patient characteristics. Approximately 59% of the children with RSV-associated hospitalizations were male. Among the 74% (*N* = 270,048) children aged < 5 years whose gestational age was known 89% (*N* = 241,625) were born at term (≥36 wGA). Among preterm infants, 16% (*N* = 4497) were extremely preterm and 56% (*N* = 16,018) moderate preterm infants. Regarding other characteristics, 3% (*N* = 11,043) had congenital heart defect, 1.2% (*N* = 4249) had bronchopulmonary dysplasia, 0.4% (*N* = 1303) had Down syndrome and < 0.1% (*N* = 166) had cystic fibrosis with pulmonary manifestations. In total, 87% (*N* = 40,009) of RSV-associated hospitalized children were otherwise healthy (median presented in Table 2).

**Table 2:** Summary of patient characteristics, seasonal median calculated over the results recorded for each RSV season (2010–2018 RSV seasons), per age group and gestational age at birth in France

	Age group		Preterm/term status ^a^	
	< 1 year old N median = 31,570	≥1 year old N median = 14,418	Preterm N median = 3616	Term N median = 31,091
**Number of males**				
Median	18,296	8838	2120	18,332
Range	15,629-19,728	7681-9554	1706-2257	12,334 - 20,051
**Comorbidities**				
*Congenital heart defect*				
Median	945	513	573	756
Range	749–1020	173–595	359–635	421–868
*Bronchopulmonary dysplasia*				
Median	304	243	495	31
Range	251–340	140–275	330–559	21–40
*Down syndrome*				
Median	106	61	18	122
Range	69–118	35–86	13–26	62–149
*Cystic fibrosis with pulmonary manifestations*				
Median	12	8	2	15
Range	6–19	5–12	0–4	8–22

*RSV* Respiratory syncytial virus

N median: median annual number of patients

^a^Only for children having determined gestational age

Over the 8-year period, 89% of RSV-associated hospitalizations were inpatient stays (at least 1 night) with a median length of stay of 3 nights. This median was 3 nights [Q1-Q3: 2–5] for children < 1 year old and 2 nights [Q1-Q3: 1–3] for children ≥1 year old. In children < 1 year old with known wGA at delivery, similar median was observed for preterm (< 36 wGA) and term children, equal to 3 nights (Table 3). Preterm children < 3 months had a median length of stay equal to 5 [Q1-Q3: 2–8] nights (Table S2). The highest average length of stay was observed in extremely preterm children (< 29 wGA) aged < 3 months (30 nights) (data not shown).

**Table 3:** Summary of the characteristics of RSV-associated hospitalizations, seasonal median calculated over the results recorded for each RSV season (2010–2018 RSV seasons), by age group and preterm status in France

	Age group		Preterm/term status ^a^	
	< 1 year old	≥1 year old	Preterm	Term
**Number of RSV patients**				
Median	31,570	14,418	3616	31,091
Range	26,885-33,952	12,606-15,485	2889-3875	21,159-34,141
**Length of RSV inpatient hospitalisation**^b^				
Median (days)	3	2	3	3
Range	2–5	1–3	2–6	2–5
**Rate of hospital stays with transition to intensive care**				
Median (%)	4.2%	0.5%	6.0%	3.0%
Range	3.3–5.2%	0.4–0.6%	5.1–7.2%	2.7–3.7%
**Number of hospital death**				
Median	6.5	3.5	1.5	8
Range	4–10	0–8	0–3	5–12
**3-month readmission rate**				
*All cause*				
Median (%)	22.9%	17.9%	35.1%	21.2%
Range	22.0–23.6%	17.1–18.9%	34.1–39.3%	20.6–22.2%
*RSV*				
Median (%)	12.7%	8.7%	21.6%	11.1%
Range	11.6–13.7%	8.2–9.2%	19.9–24.6%	10.4–11.8%

*RSV* Respiratory syncytial virus

^a^Only for children having determined gestational age

^b^ Calculated overall RSV seasons

Three percent of RSV-associated hospitalizations (*N* = 12,496 stays) were followed by an admission to intensive care unit (ICU). Around 95% of these ICU stays were reported in children < 1 year old and more than half were observed in term children (Table 3). The median rate of RSV-associated hospitalizations with ICU admission was 4.2% [range 3.3–5.2%] for children < 1 year old and 0.5% [range 0.4–0.6%] for children ≥1 year old. Children < 3 months recorded a higher median rate of RSV-associated hospitalizations with ICU admission equal to 7% [range 6.2–8.8%] (Table S2).

A small number of deaths were recorded during RSV-associated hospitalizations, corresponding to less than 13 deaths by RSV season and around two third of t these children had multiple risk factors (68%) (Table 3). The risk of in-hospital death remained constant during the study period.

Twenty-one percent of children were hospitalized (all-causes considered) in the 3 months following the initial RSV-associated hospitalization, of which two third (64%) were < 1 year old. Twelve percent of children were hospitalized for another RSV episode in the 3 months following the initial RSV-associated hospitalization, this rate was two times higher for the preterm (21.6%) compared to term children (11.1%) (Table 3).

### Economic burden of RSV

**Fig. 3:**
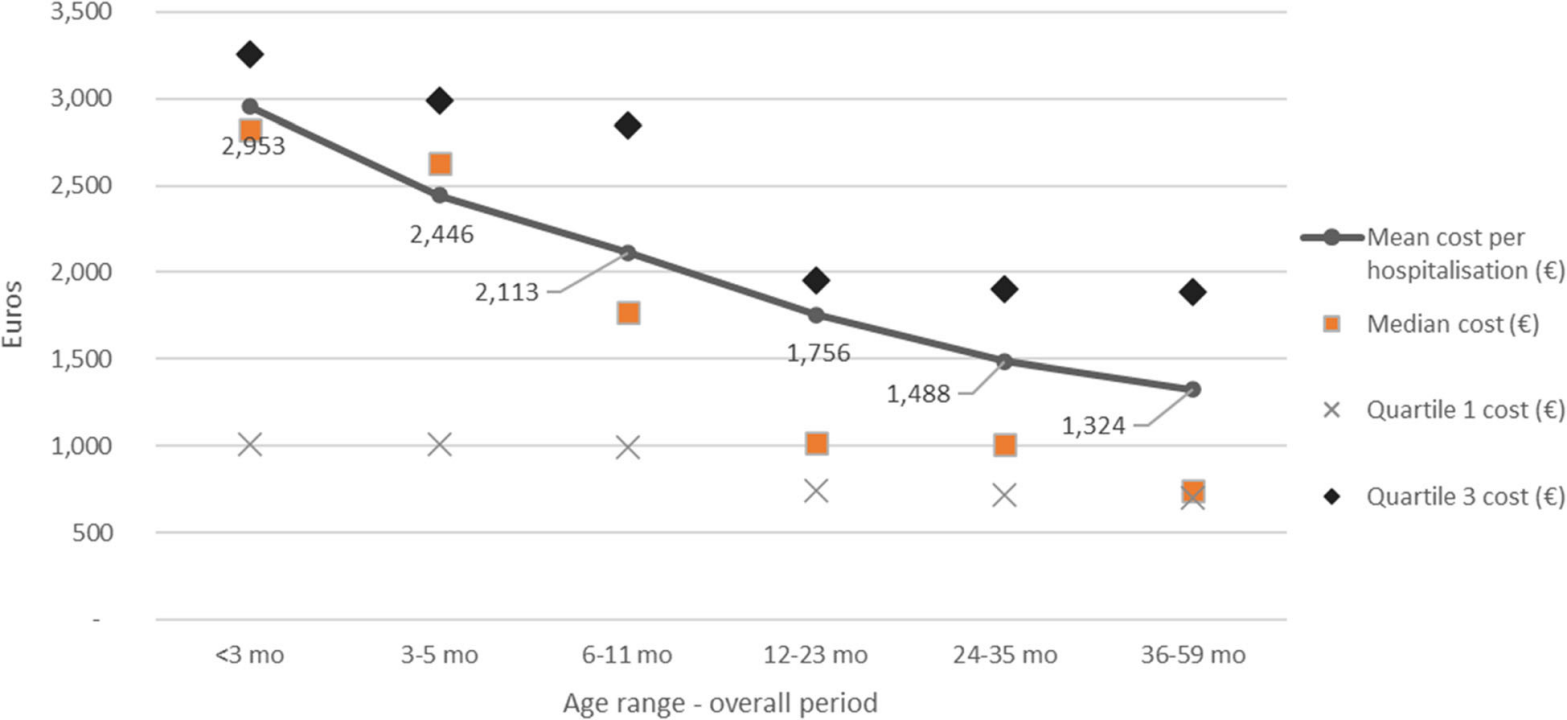
RSV associated hospitalization cost distribution by age group during RSV seasons from 2010 through 2018 in France

Over the 8-year period the total direct cost of RSV-associated hospitalizations was estimated at €931.6 million (Table 4). Annual costs increased between 2010 and 11 and 2017–18, from €93.2 million to €124.1 million, consistently with the growing number of RSV-associated hospitalizations. The average cost of RSV-associated hospitalization, estimated at €2289 (SD 2114) in children aged < 5 years old, decreased with age, being 2 times higher for infants aged of < 3 months compared to the oldest children (36–59 months) (Fig. 3). In children < 1 year old, the mean cost was estimated at €2607 (SD 2317). Irrespective of the season, RSV-associated hospitalizations in children < 1 year old represented almost 80% of the total cost, of which half was generated by hospital stays of infants < 3 months. The average RSV-associated hospitalization cost of a child with at least one risk factor was estimated at €2947 (SD 3809) compared to €2208 (SD 1781) for children without risk factor (data not shown). RSV-associated hospitalization of term children generated 66% of the total cost, from €52.5 million in 2010/2011 to €85.6 million euros in 2017/18 (considering term and unknown wGA children, it represents 89% of the total cost: €83.3 million in 2010/11 and 110.2 million in 2017/18).

**Table 4:** RSV total hospitalization cost by age range during RSV periods between 2010 and 2018 in France

			Age Range (month old)				
RSV season	< 3 mo	3–5 mo	6–11 mo	12–23 mo	24–35 mo	36–59 mo	Total
2010/2011	37,006,869 €	18,373,618 €	16,320,666 €	12,072,373 €	4,554,388 €	4,847,626 €	93,175,540 €
2011/2012	41,167,182 €	20,126,198 €	18,320,858 €	13,393,522 €	5,029,325 €	4,952,583 €	102,989,668 €
2012/2013	49,873,208 €	21,415,497 €	17,307,913 €	13,152,263 €	5,440,949 €	5,839,329 €	113,029,159 €
2013/2014	47,793,614 €	22,214,521 €	17,390,682 €	12,586,276 €	5,152,541 €	5,555,681 €	110,693,315 €
2014/2015	44,380,454 €	19,406,961 €	16,817,894 €	12,519,617 €	5,881,104 €	6,994,634 €	106,000,664 €
2015/2016	62,859,377 €	27,329,493 €	20,818,388 €	14,531,938 €	5,786,835 €	6,101,165 €	137,427,195 €
2016/2017	64,312,635 €	29,300,573 €	22,819,097 €	15,643,755 €	6,059,861 €	6,030,519 €	144,166,440 €
2017/2018	54,565,966 €	24,924,750 €	19,856,450 €	13,186,120 €	5,538,018 €	6,039,978 €	124,111,282 €
Mean annual cost	50,244,913 €	22,886,451 €	18,706,494 €	13,385,733 €	5,430,378 €	5,795,189 €	116,449,158 €

Among all RSV-associated hospitalizations, 330 (0.1%) corresponded to birth hospitalizations. The total cost of these stays represented €6.7 million euros (0.7% of the total costs estimated within the 2010–2018 period). After excluding these stays, the average cost of RSV-associated hospitalization in infants aged < 3 months old was estimated at €2911 (SD 2388) and the total cost of RSV-associated hospitalizations over the study period was estimated at €924 million for all age groups and at €728 million euros for the children < 1 year old, corresponding to an average annual cost of €91 million. The total cost of stays for children < 1 year old coded J45/R062 was estimated at €28.7 million (average annual cost €3.6 million), representing 3.9% of the total cost for children < 1 year old estimated over the entire study period.

## Discussion

This study is the first to describe the national burden of RSV-associated hospital admissions in children aged < 5 years in France during a large time period (2010–2018). The incidence rate of RSV-associated hospitalization increased over time from 1.3 to 2.4 per 1000 person-months. Children < 1 year old were the most impacted by RSV-associated hospitalizations as they represent 69% of the hospitalizations, with associated high costs. We observed a correlation between month of birth and RSV-associated hospitalization and an equal distribution of RSV-associated hospitalization from infants born outside or during the RSV season.

Since the virological test is not systematically requested, the decision to include J219: “acute bronchiolitis, unspecified” in addition to RSV specific ICD-10 are J210, J121 and J205 was made to limit the under-estimation of RSV-hospitalizations. This observation was highlighted by Cai et al. who showed that RSV-specific ICD-10 codes had a sensitivity of 14% Adding J219 in our analysis increased the sensitivity without compromising the specificity [17]. Furthermore, a recent laboratory surveillance report from Lyon hospital centres (HCL, France) showed that the incidence of hospitalization with a confirmed RSV in the first 3 months of life for children between September and November was equal to 3.45% (unpublished data). This result is close to our estimation (incidence rate 3.37%, same age and year) which validates the ICD-10 codes selected for this study.

The rising trend of incidence over time has already been described in other studies, particularly in the United Kingdom [18, 19]. According to the authors, this observed increase was unlikely due to changes in disease incidence or severity, but mostly reflected the increase in hospital admissions observed for the last 15 years [20]. This could be attributed to the lowering of hospital admission thresholds and the decline in management of acute outcomes at the community. In France over the last 15 years, a 45% increase of outpatient visits has been observed in hospital setting [21]. A complementary analysis of our data showed that the incidence of RSV hospital outpatient visit and the incidence of RSV hospital inpatient stays have increased during the 8-year period, respectively from 0.19 to 0.28 per 1000 person-months and 1.6 to 2.1 per 1000 person-months (Table S4). Changes in air pollution could have contributed to this increasing of RSV-associated hospitalization incidence. A 2018 study in Paris showed that winter air pollution was positively associated with both outpatient visits and hospitalizations in the occurrence of acute severe bronchiolitis [22].

In our study, we observed a negative correlation between RSV-associated hospitalization incidence and age groups, most of the burden being observed among children < 1 year old. This trend was also described by other studies [14, 18, 19]. Our findings, from 37 to 53 RSV hospitalizations per 1000 person-years in infants < 1 year of age between 2010 and 2018, is in accordance with the French study conducted using the PMSI database in 2009 with a bronchiolitis-associated hospitalization rate of 35.8 per 1000 infants [10]. RSV-associated hospitalization rates were the highest for children born between September and November and early exposed to their first RSV season. This result has been reported in previous studies such as the study from Kramer et al. [11], where the highest risk of RSV-associated hospitalization was observed for children born in November. Lloyd et al. reported the highest risk of RSV-associated hospitalization in the first year of life for children born between October and February with a peak in December followed by a decrease until April [24]. In our study, the risk of RSV-associated hospitalization decreased from November to March, which can be explained by maternal exposure to RSV during pregnancy during the early months of the winter epidemic. The transfer of maternal RSV antibodies to the foetus then confers protection of the child against the virus after birth [25, 26]. In our results we also show a similar number of RSV-associated hospitalizations, between infants born outside or during RSV season exposed to their first RSV season. The same observation could be derived from studies by Lloyd et al. and Reeves et al. [24, 27]*.* This finding may support RSV immunization of all infants before the RSV season, as discussed by Janet et al. [28].

Regarding the economic burden of RSV, the average annual cost due to RSV hospitalizations was estimated at €116 million, with a mean cost per stay of €2289, of the same order of magnitude as that reported in the Danish study (around €1830) [23]. Similar to the number of hospitalizations, the total cost increased over the time period, mainly among infants < 1 year that represents 80% of the total cost of hospitalizations in our study. Hospital admissions of preterm children (7% of all RSV hospitalizations) generated 11% of the total costs. These elements echo the results of another study which evaluated the long-term RSV burden among pre-term and term infants in the US, that also reported prematurity and young age group (< 1 year old) as risk factors linked to high economic burden [29]. In comparison, the French study from Kramer showed a higher hospitalization cost in children < 1 year old compared to our results illustrating the cost heterogeneity across the country [11].

One limitation of our study, was the inclusion of asthma and wheezing ICD-10 codes, non-specific of RSV infections. Interestingly, asthma became the main cause of hospital admission in children ≥1 year old during the epidemic months (50% of hospitalisations among children aged between 1 and 2 years old and 90% in children> 2 years old). This observation is in agreement with other studies [5], and most probably because children ≥1 year old are not systematically tested for RSV. Therefore, the virus involved is not always mentioned in hospital discharges, leading to underestimate the RSV hospitalization burden when RSV specific codes are used alone. Moreover, we observed a similar temporal distribution of asthma vs RSV-associated hospitalization codes (data not shown) during the RSV season and especially at its peak, making asthma related hospitalization a good proxy for RSV hospitalization in older children.

## Conclusion

This study describes the high burden of RSV-associated hospitalizations in French children and mostly in the < 1 year old population with an associated average annual cost of €91 million. During the last RSV season studied 2017–18, RSV-associated hospitalizations alone accounted for 28% of all-cause hospitalizations among children < 1 year old. We also demonstrated healthcare system pressure in France every winter due to RSV-hospitalizations from infants born inside and outside their first RSV season. This approach could be complemented by RSV burden estimates in community setting or using ecological approach to estimate hospitalization attributable to RSV using excess modelling method [30, 31].

## Supplementary Information

**Additional file 1: Table S1**. Number of all-causes hospitalizations and percentage of RSV associated hospitalizations among all-causes hospitalizations by age group and type period (RSV season and year (July–June)), from 2010 to 2018 in France**. Table S2**. Summary of the characteristics of the hospitalizations for RSV infection for < 3 months old group, median indicator calculated over the indicators recorded for each RSV season (2010–2018 RSV seasons), total and per gestational age at birth in France. **Table S3**. Incidence of RSV hospitalizations during the respiratory year (July N – June N + 1) between 2010 and 2018 (per 1000 person-years). **Table S4**. Rate of RSV-related hospital inpatient/outpatient stays per 1000 person-months. **Fig. S1**. Breakdown of RSV-associated hospitalizations by RSV-specific ICD-10 codes among primary and associated diagnoses, stratified by age group from 2010 through 2018 in France.

## Data Availability

All data generated or analysed during this study are included in this published article and its supplementary files. The data that support the findings of this study are available from the Technical Agency for Information on Hospital Care - ATIH (PMSI holder). But restrictions apply to the availability of these data, which were used with an authorization for the current study, and so are not publicly available.
