# Correction: Economic and disease burden of RSV-associated hospitalizations in young children in France, from 2010 through 2018

**DOI:** 10.1186/s12879-023-08049-7

**Published:** 2023-02-27

**Authors:** C. Demont, N. Petrica, I. Bardoulat, S. Duret, L. Watier, A. Chosidow, M. Lorrot, A. Kieffer, M. Lemaitre

**Affiliations:** 1 Sanofi Pasteur, 14 Espace Henry Vallée, 69007 Lyon, France; 2 IQVIA, 92400 Courbevoie, France; 3 Université Paris-Saclay, UVSQ, Inserm, CESP, 94807 Villejuif, France; 4 Department of Pediatrics, Armand Trousseau Hospital (AP-HP), 75012 Paris, France

**Correction** **:** **BMC Infectious Diseases (2021) 21:730** 10.1186/s12879-021-06399-8

Following publication of the original article [1], the authors would like to correct the average number of hospitalizations shown in the results section within the abstract.

- The sentence currently reads:
- “On average 45,225 RSV-associated hospitalizations…”
- The sentence should read:
- “On average 50,878 RSV-associated hospitalizations…”

The original article [1] has been corrected.
