# Supplementary material for: Economic and disease burden of RSV-associated hospitalizations in young children in France, from 2010 through 2018
Source: BMC Infect Dis. 2021 Aug 2;21:730. doi: 10.1186/s12879-021-06399-8 (PMC8327424; doi:10.1186/s12879-021-06399-8)
Supplement: Supplementary file 1 — Additional file 1: Table S1. Number of all-causes hospitalizations and percentage of RSV associated hospitalizations among all-causes hospitalizations by age group and type period (RSV season and year (July–June)), from 2010 to 2018 in France. Table S2. Summary of the characteristics of the hospitalizations for RSV infection for < 3 months old group, median indicator calculated over the indicators recorded for each RSV season (2010–2018 RSV seasons), total and per gestational age at birth in France. Table S3. Incidence of RSV hospitalizations during the respiratory year (July N – June N + 1) between 2010 and 2018 (per 1000 person-years). Table S4. Rate of RSV-related hospital inpatient/outpatient stays per 1000 person-months. Fig. S1. Breakdown of RSV-associated hospitalizations by RSV-specific ICD-10 codes among primary and associated diagnoses, stratified by age group from 2010 through 2018 in France. [file 12879_2021_6399_MOESM1_ESM.docx]

**Economic and disease burden of RSV-associated hospitalizations in young children in France, from 2010 through 2018**

C Demont^1^, N Petrica^2^, I Bardoulat^2^, S Duret^2^, L Watier^3^, A Chosidow^4^, M Lorrot^4^, A Kieffer^1^, M Lemaitre^2^

1 Sanofi Pasteur, 69007 Lyon, France ;

2 IQVIA, 92400 Courbevoie, France ;

3 Université Paris-Saclay, UVSQ, Inserm, CESP, 94807 Villejuif, France ;

4 Department of Pediatrics, Armand Trousseau Hospital (AP-HP) 75012 Paris, France

**Word count:** 3,487 words

**Version** 9 February 2021

**Supplement material**

Table S1. Number of all-causes hospitalizations and percentage of RSV associated hospitalizations among all-causes hospitalizations by age group and type period (RSV season and year (July-June)), from 2010 to 2018 in France

|  | <1 year old | | | | ≥1 year old | | | |
| --- | --- | --- | --- | --- | --- | --- | --- | --- |
|  | RSV season | | Year (July-June) | | RSV season | | Year (July-June) | |
|  | N (all-causes hospitalizations) | % of RSV | N (all-causes hospitalizations) | % of RSV | N (all-causes hospitalizations) | % of RSV | N (all-causes hospitalizations) | % of RSV |
| 2010/2011 | 134,687 | 22.2 | 235,893 | 12.7 | 268,307 | 5.1 | 512,423 | 2.7 |
| 2011/2012 | 135,267 | 24.3 | 234,713 | 14.0 | 269,844 | 5.6 | 509,537 | 3.0 |
| 2012/2013 | 137,739 | 25.6 | 239,200 | 14.7 | 261,004 | 6.0 | 500,939 | 3.1 |
| 2013/2014 | 136,387 | 25.9 | 236,487 | 14.9 | 253,064 | 6.0 | 489,254 | 3.1 |
| 2014/2015 | 138,188 | 24.5 | 239,071 | 14.2 | 258,915 | 6.5 | 491,995 | 3.4 |
| 2015/2016 | 140,056 | 27.3 | 241,323 | 15.8 | 254,050 | 6.5 | 489,550 | 3.4 |
| 2016/2017 | 139,522 | 27.0 | 238,117 | 15.8 | 254,944 | 6.5 | 483,487 | 3.4 |
| 2017/2018 | 139,741 | 27.7 | 238,489 | 16.2 | 248,187 | 6.4 | 467,847 | 3.4 |
| Mean annual cost | 137,698 | 25.6 | 237,912 | 14.8 | 258,539 | 6.1 | 493,129 | 3.2 |

Table S2. Summary of the characteristics of the hospitalizations for RSV infection for <3months old group, median indicator calculated over the indicators recorded for each RSV season (2010-2018 RSV seasons), total and per gestational age at birth in France

|  | <3 months old group | | |
| --- | --- | --- | --- |
|  | Total** | Preterm* | Term* |
| **Number of RSV patients** | | | |
| Median | 15,360 | 820 | 11,377 |
| Range | 12,209 - 16,498 | 737-829 | 8,565-12,507 |
| **Length of RSV inpatient hospitalisation** | | | |
| Median (age) | 4 | 5 | 4 |
| [Q1-Q3] | 2-6 | 2-8 | 2-6 |
| **Rate of hospitalizations with admission to the intensive care unit** | | | |
| Median (%) | 7.0% | 1.1% | 5.0% |
| Range | 6.2%-8.8% | 0.97% -1.2% | 4.0%-6.3% |
| **Number of hospital death** | | | |
| Median | 3 | 1 | 2 |
| Range | 3-5 | 1-2 | 1-4 |
| **3-month readmission rate** | | | |
| *All cause* |  |  |  |
| Median (%) | 25.2% | 46.2% | 25.5% |
| Range | 23.8%-26.3% | 43.4% -47.2% | 24.5%-26.7% |
| *RSV-associated* |  |  |  |
| Median (%) | 13.5% | 28.1% | 13.3% |
| Range | 12.1%-15.3% | 27.0%-30.6% | 11.9%-15.1% |
| RSV: Respiratory syncytial virus | | | |
| *Only for children having determined gestational age | | | |
| ** Children with and without determined gestational age | | | |

Table S3. Incidence of RSV hospitalizations during the respiratory year (July N – June N+1) between 2010 and 2018 (per 1,000 person-years)

|  | Age Range (months old) | | | | | | |
| --- | --- | --- | --- | --- | --- | --- | --- |
| Respiratory year | <3 mo | 3-5 mo | 6-11 mo | 12-23 mo | 24-35 mo | 36-59 mo | Total |
| 2010/2011 | 69 | 41 | 20 | 9 | 4 | 2 | 11 |
| 2011/2012 | 77 | 44 | 22 | 10 | 4 | 2 | 12 |
| 2012/2013 | 90 | 47 | 22 | 10 | 5 | 3 | 13 |
| 2013/2014 | 88 | 48 | 22 | 9 | 4 | 3 | 13 |
| 2014/2015 | 86 | 45 | 22 | 10 | 5 | 3 | 13 |
| 2015/2016 | 100 | 53 | 24 | 10 | 5 | 3 | 14 |
| 2016/2017 | 98 | 55 | 25 | 11 | 5 | 3 | 14 |
| 2017/2018 | 104 | 58 | 26 | 10 | 5 | 3 | 14 |

Table S4. Rate of RSV-related hospital inpatient/outpatient stays per 1 000 person-months

| **RSV season** | **2010/11** | **2011/12** | **2012/13** | **2013/14** | **2014/15** | **2015/16** | **2016/17** | **2017/18** |
| --- | --- | --- | --- | --- | --- | --- | --- | --- |
| Inpatient stay | 1.6 | 1.8 | 1.9 | 1.9 | 1.9 | 2.1 | 2.1 | 2.1 |
| Outpatient stay | 0.19 | 0.21 | 0.21 | 0.21 | 0.22 | 0.26 | 0.27 | 0.28 |

Figure S1. Breakdown of RSV-associated hospitalizations by RSV-specific ICD-10 codes among primary and associated diagnoses, stratified by age group from 2010 through 2018 in France
